# Occupational Deaths among Healthcare Workers

**DOI:** 10.3201/eid1107.041038

**Published:** 2005-07

**Authors:** Kent A. Sepkowitz, Leon Eisenberg

**Affiliations:** * Memorial-Sloan-Kettering Cancer Center, New York, New York, USA;; † Harvard Medical School, Boston, Massachusetts, USA

**Keywords:** Hepatitis, HIV, SARS, tuberculosis, healthcare worker, occupational safety, vaccinia

## Abstract

Recent experiences with severe acute respiratory syndrome and the US smallpox vaccination program have demonstrated the vulnerability of healthcare workers to occupationally acquired infectious diseases. However, despite acknowledgment of risk, the occupational death rate for healthcare workers is unknown. In contrast, the death rate for other professions with occupational risk, such as police officer or firefighter, has been well defined. With available information from federal sources and calculating the additional number of deaths from infection by using data on prevalence and natural history, we estimate the annual death rate for healthcare workers from occupational events, including infection, is 17–57 per 1 million workers. However, a much more accurate estimate of risk is needed. Such information could inform future interventions, as was seen with the introduction of safer needle products. This information would also heighten public awareness of this often minimized but essential aspect of patient care.

The fundamental ethic of health care is that sick persons must receive care (1). This premise carries an unstated consequence: an occupational risk to healthcare workers who respond to the needs of contagious patients. This predicament was shown yet again during the severe acute respiratory syndrome (SARS) epidemic. As often occurs when infectious disease outbreaks are caused by an emerging agent, healthcare workers were the group most affected. According to the World Health Organization, 8,098 cases occurred during the outbreak, and 774 (9.6%) persons died (2). Healthcare workers accounted for 1,707 (21%) of the cases (2).

More specific information from outbreak hospitals in Hong Kong (3), Singapore (4), Guangdong Province (5), and Toronto (6,7) showed that 378 (57%) of 667 cases occurred in healthcare workers or medical students. The higher proportion in these reports may be attributable to the availability of more detailed site-specific information. The number of fatal infections in healthcare workers is not known, but deaths have been reported.

Of course, SARS is not the only infection that presents an occupational risk to healthcare workers. During the past 2 decades, occupationally acquired hepatitis B, HIV infection, multidrug-resistant tuberculosis, and viral hemorrhagic fevers, among others, have killed healthcare workers. In earlier generations, diseases such as occupationally acquired tuberculosis, measles, diphtheria, and scarlet fever posed substantial risk (8,9). In response, the Centers for Disease Control and Prevention (CDC) and other organizations have promulgated guidelines for healthcare worker protection, recommending vaccination, early patient screening, isolation precautions, and use of personal protective equipment (10). Perhaps the most successful is the 1991 Occupational Safety and Health Administration (OSHA) bloodborne pathogen standard, which contributed to reduction of hepatitis B among healthcare workers (11).

Despite this recognized risk, no country has a system in place to track fatal, occupationally acquired infections in their entirety. In this article, we examine occupational death rates for healthcare workers by using currently available US federal data sources. To provide more inclusive rates, we also estimate the number of annual deaths from occupationally acquired infections.

## Methods

### Available Data: Numerator

The US Department of Labor, through the Bureau of Labor Statistics, maintains an annual "census of fatal occupational injuries" across a wide range of occupations and exposures as part of its injuries, illness, and fatalities program (12). Federal law compels employers to notify OSHA of any occupational death within 8 hours of the death by telephone or in person at a local OSHA office (13). OSHA then reports the data in 2 ways: by occupation or by industry. When classified by "occupation," healthcare workers are placed into any of 7 broad groups. Physicians and nurses, for example, are categorized as "managerial and professional specialty," while health technologists and technicians are grouped under "technical, sales, and administrative support," and nursing aides, orderlies, and attendants are considered "service occupations."

In contrast, the "industry" classification classifies all healthcare workers into "health services" without additional job-specific information. The annual death totals derived from "occupation" and from "industry" classifications differ by ≈15%–20%.

In either approach, OSHA places all deaths into 1 of 6 distinct categories: transportation accidents, assaults and violent acts, contact with objects and equipment, falls, exposure to harmful substances or environments, and fires and explosions. Because deaths from occupationally acquired diseases such as tuberculosis or hepatitis are not routinely captured in this system, the occupational risk of healthcare work is underestimated (12).

Although no national agency systematically tracks deaths due to occupationally acquired infection, both percutaneous injuries and tuberculin skin test conversions are reported to OSHA by completing the OSHA Form 300 (Log of Work Related Injuries and Illnesses), OSHA Form 301 (Injury and Illness Incident Report), or both. The latter requires more specific information about how the injury or illness occurred. The number of unreported events is not known; however, an institution may be cited or fined for incomplete records, which probably improves compliance.

Needlestick-related deaths are only occasionally reported through this system. According to OSHA data, from 1992 to 2002, a total of 67,363 workers died of occupational injuries, including 28 healthcare workers who died of complications related to needlestick exposures. OSHA cautions, however, that they collect and report fatal work injuries; needlestick data therefore reflect only those cases that fall within the 6 defined injury definitions (K. Loh, pers. comm.).

The National Institute for Occupational Safety and Health (NIOSH), a branch of CDC, is charged with providing leadership and conducting research to prevent workplace illness and injury. They regularly publish the Worker Health Chartbook, which reports fatal occupational illnesses (14). Infectious diseases, however, are not included in the illness report. Instead, data are focused on occupational pneumoconiosis, mesothelioma, and hypersensitivity pneumonitis.

NIOSH information regarding occupational infection is derived from 4 federal health databases as "nonfatal illnesses" (14). These databases include the National Surveillance System for Healthcare Workers, which obtains information from 60 hospitals that voluntarily submit needlestick and tuberculin conversion data on a regular basis. The Viral Hepatitis Surveillance Program and the Sentinel Counties Study of Acute Viral Hepatitis track incident cases of hepatitis, including those occurring in healthcare workers. Cases of AIDS and HIV infection among healthcare workers are gathered from several sources, including the CDC HIV/AIDS Surveillance Reporting System. Finally, staffTRAK-TB is used by tuberculosis control programs to monitor skin test conversion rates.

These data sources, although useful, have substantial limitations. First, they measure only the initial injury or exposure and not the consequent disease. Most needlesticks and tuberculin conversions do not result in disease; rarer yet are those that lead to fatal infection. Thus, rates of needlestick and tuberculin conversions, although meaningful, may not accurately reflect the outcomes of greatest interest: disease and death. Further complicating this problem, the latent period from initial infection to disease for HIV, tuberculosis, and other infections is measured in years to decades. For example, a worker may sustain a needlestick, become infected with HIV, but not develop clinical symptoms for several years. In the interval, the worker may have changed jobs several times, making linking the exposure to the disease difficult.

In addition, the tuberculin skin test is notoriously difficult to interpret, with suboptimal sensitivity and specificity, and so may distort the actual trend in tuberculosis infection rates. Finally, as many as 50%, and possibly more, of all percutaneous injuries are not reported, which complicates tracking by the current passive surveillance system (15).

### Available Data: Denominator

To calculate an annual occupation-specific fatality rate, we determined the number of persons at risk per occupation by using 2 datasets from the Department of Labor: the 2001 National Occupational Employment and Wage Estimates (16) and the 2002 Current Population Survey (CPS) (17). A major difference between these 2 data sources is the inclusion of self-employed workers in the CPS report. In addition, only the CPS counts experienced but unemployed workers.

## Results

The US labor force is composed of 136 million persons, 6 million of whom are healthcare workers with potential patient contact (16,17). Approximately half of these are registered or licensed practical nurses. An additional 3 million persons work in healthcare-support occupations and may have patient contact, including nursing aides, orderlies, and attendants (1.3 million); home health aides (560,000); and medical or dental assistants (600,000) (16,17). These estimates do not include persons without routine patient contact employed in such occupations as healthcare administrators, medical secretaries, and other clerical staff (16,17).

From 2000 to 2002, the Department of Labor reported an annual average of 77 healthcare worker deaths with the "industry" categorization versus 93 deaths with the "occupation" category (12). Deaths from transportation accidents and assaults and violent acts accounted for most. To address the latter problem, NIOSH recently published the monograph Violence: Occupational Hazards in Hospitals (http://www.cdc.gov/niosh/2002-101.html).

Specific data are reported for some but not all healthcare worker occupations in the "occupation" classification (Table 1). For example, an annual average of 10 doctors, 18 registered nurses, and 18 health technologists/technicians died. In addition, statistics maintained by the National EMS (Emergency Medical Services) Memorial Service show that ≈12 emergency medical service workers are killed annually, including 13 in 2002 (18). The EMS deaths are not specifically noted in the Department of Labor statistics; therefore, whether these deaths are included in the overall number is uncertain.

**Table 1:** Occupation-specific death rates for US healthcare workers*

Occupation	Number employed (× 10^3^)	Total deaths	Death rate
Emergency medical services	116–170	11	64–95
Physicians	340–820	10	12–29
Registered nurses	2,300	18	8
Technologists and technicians	650	18	28
Home nursing aides, orderlies, and attendants	1,700	13	8

*Rates expressed per 1 million workers. Numbers reflect 3-year average (2000–2002) of violent deaths and do not include infectious causes. Emergency medical services deaths reflect 4-year average (1999–2002) and exclude deaths sustained in the collapse of the World Trade Center towers in 2001. Range of number employed reflects 2 different federal databases (see text) (12,13,16,17).

### Estimated Deaths from Specific Infections

To estimate the contribution of occupationally acquired infection, we examined the effects of hepatitis B, hepatitis C, HIV infection, and tuberculosis by using available information on disease incidence and natural history. Table 2 combines both injury-related data reported to the Department of Labor (shown in Table 1) and our estimates from specific infections, detailed below. Overall, we estimate that 9–42 healthcare workers per million die annually from occupational infection.

**Table 2:** Occupational deaths among US healthcare workers (HCW), 2002*

Cause of death	No. deaths	HCW death rate, excluding support occupations (N = 6.2 million)	HCW death rate, including support occupations (N = 9.1 million)
Injury	77–93	12–15	8–10
Infection-related†	80–260	13–42	9–29
Total	157–353	25–57	17–39

*Rates expressed per 1 million workers. Estimates based on incidence and natural history of specific infections. Number of deaths by injury reflect 3-year average (2000–2002) (12,13,16,17). †Includes deaths from hepatitis B virus (75–250) and hepatitis C virus, HIV, and tuberculosis (5–10 total).

## Hepatitis B

CDC estimates that, in 1983, 10,000 healthcare workers became infected with hepatitis B through occupational exposure (M.J. Alter, pers. comm.). The natural history of hepatitis B infection indicates that chronic infection developed in 5%–10% (500–1,000) of these persons. Although estimates vary, as many as 15%–25% (75–250 persons) of those with chronic infection will die from a hepatitis B–related complication, including cirrhosis or hepatocellular carcinoma (19). Since the time from infection to serious medical disease in the subset with these complications typically is about 20 years, most of these deaths can be expected to occur during this decade.

The risk of hepatitis B has diminished by >90% since the introduction of standard precautions and a recombinant vaccine (11). Despite vaccine availability, however, coverage is incomplete because >30% of workers refuse to be vaccinated (11). As a consequence, CDC estimates that, in 2002, another 400 healthcare workers became infected with hepatitis B virus, a number that has been stable since 1995 (M.J. Alter, pers. comm.).

## Hepatitis C

CDC estimates that 3.9 million persons in the United States, or 1.8% of the population, have been infected with hepatitis C virus and that 2.7 million (1.3%) are chronically infected (20,21). Healthcare workers as a group have the same hepatitis C virus seroprevalence as the rest of the US population (20). However, transmission from a hepatitis C–infected patient to a healthcare worker occurs in 1%–3% of percutaneous exposures (22). With an estimated 380,000 percutaneous injuries annually (23), 50–150 transmissions would be expected, assuming that hospitalized patients have the same hepatitis C virus seroprevalence as the rest of the US population. Our understanding of the natural history of hepatitis C virus (HCV) continues to evolve; however, as many as 5% of those infected, or 3–8 healthcare workers annually, can be expected to die of liver disease.

This estimate may be low because hospitalized patients in some regions may have rates of HCV infection well above that of the US population, raising the likelihood of exposure to a positive source case. HCV seroprevalence of at least 5% has been reported in several groups who are frequently hospitalized. These groups include patients requiring dialysis (20), intravenous drug users with or without HIV infection (20), and perhaps patients in psychiatric hospitals and outpatient facilities (24).

## Human Immunodeficiency Virus

CDC distinguishes between "documented" and "possible" occupational transmission of HIV. Documented infection refers to documented seroconversion in healthcare workers after occupational exposure or other laboratory evidence of occupational infection. Possible infection refers to history of occupational exposure to infected blood or other fluid in healthcare workers without identifiable behavioral or transfusion risks, but for whom seroconversion specifically resulting from an occupational exposure was not documented, i.e., a baseline, postexposure test for HIV was not performed (25).

To date, 26 (46%) of 57 US healthcare workers with voluntarily reported, documented, occupationally acquired HIV infection have progressed to AIDS, as have 121 (88%) of 138 healthcare workers with possible occupational transmission (25). Job-specific information is available for persons with either documented or possible disease. Twenty-four (42%) of 57 proven transmissions have occurred in nurses, 16 (28%) in clinical laboratory technicians, and 6 (11%) in nonsurgical physicians. Among the 138 persons with possible occupational acquisition, in addition to the occupations above, cases were noted among 12 emergency medical technicians (9%), 6 surgeons (4%), 15 health aide/attendants (11%), and 13 housekeepers and maintenance workers (9%). This distribution by occupation may be applicable to other infections transmitted by percutaneous injury, such as hepatitis B and hepatitis C, but comparable information from recent studies of these infections is not available.

Antiviral therapy to manage an occupational exposure to HIV has resulted in severe hepatitis requiring liver transplant, though no therapy-related deaths have been reported (26). The number of healthcare workers who have died from proven or probable occupationally acquired HIV infection has not been reported, but some have died and risk for serious complication persists (27).

## Tuberculosis

Tuberculosis has long represented an occupational threat to healthcare workers (28). This risk became particularly evident during the late 1980s and early 1990s, when several nosocomial outbreaks of multidrug-resistant tuberculosis occurred in the United States. During these years, workers experienced a tuberculin conversion, and several developed active drug-resistant disease (29). The infection was fatal in at least 9 immunocompromised healthcare workers (30). Treatment for occupationally acquired resistant tuberculosis also has resulted in death (31). Healthcare workers who became latently infected with multidrug-resistant tuberculosis strains at this time remain at risk for disease reactivation (32).

In 2003, CDC and the American Thoracic Society revised the recommendation for treatment of latent *Mycobacterium tuberculosis* infection with pyrazinamide and rifampin because of an unexpectedly high rate of hepatotoxicity with this regimen (33). According to CDC, 6 healthcare workers were included in the 49 persons in whom severe hepatitis developed or death occurred. One healthcare worker died from this complication (K. Ijaz, pers. comm.).

## Occupational Death Rate among Other Workers

To place healthcare worker risk into context, we applied the same approach to derive average annual death rates among several worker groups for the 3-year period 2000–2002 (Table 3). The US workforce has a rate of ≈41 occupational fatalities per million workers. Fishermen and construction workers have the highest rate (>1,000 deaths per million workers annually). Members of the military (361–671 per million), police and related protective service workers (108 per million), and firefighters (93 per million) also have markedly elevated rates. Lawyers (7–14 per million) and waiters (5 per million) have relatively low rates of occupational death.

**Table 3:** Occupational death rate for various jobs, United States (in descending order)*

Occupation	No. employed (× 10^3^)	Total deaths	Death rate
Fisherman	39	46	1,179
Construction worker	825–1,108	1,198	1,081–1,452
Pilot	107–129	102	791–953
Military (active and reserve)	2,600	94	361
Truck driver	2,544–3,365	530	157–208
Protective service	2,000	219	108
Firefighter	1,100	102	93
US workforce	136,000	5,780	42.5
Healthcare worker	6,200–9,100	157–353	17–57
Sheetmetal worker	172–207	8	39–46
Bartender	339–427	10	23–29
Lawyer	490–920	6	7–14
Waiter	1,893–1,981	9	5

*Numbers represent average of annual deaths during 3-year period, 2000–2002. Range of number employed reflects 2 different federal databases (see text). Rates expressed per 1 million workers (12,13,16,17).

## Infectious Risk to Healthcare Workers Internationally

The risk of acquiring a work-related fatal infection represents a substantial risk to healthcare workers in developing countries (34). In addition to viral hemorrhagic fevers, occupationally acquired tuberculosis in Africa is increasingly recognized. Reports from Malawi (35), Ethiopia (36), and South Africa (37) describe substantial rises in active cases of tuberculosis among healthcare workers, many of whom die of the disease (25% in a series from Malawi). Few reports have examined the occupational risk for HIV infection in disease-endemic, resource-poor countries, but transmission is likely (38).

## Summary and Recommendations

We estimate that 17–57 US healthcare workers per million employed die annually from occupational infections and injuries (Table 2). However, the number of deaths that results from occupationally acquired infection is an educated guess at best. We projected the potential consequences of only 4 diseases by relying on the published prevalence, transmission rate, and natural history of these infections. Our results therefore may underestimate the actual occupational death rate for these diseases. Furthermore, these estimates do not account for deaths from other infections, which demonstrates the problems engendered by the current lack of a national tracking system. This finding stands in contrast to the rigorous approach used to track occupational deaths of various other workers, such as police officers and firefighters (39,40).

The recent experiences with SARS and smallpox vaccination have demonstrated the vulnerability of healthcare workers to occupationally acquired infections. In addition, these events have served as a reminder of the critical societal responsibility of the healthcare worker. Although not as central to a national disaster response as protective service workers, healthcare workers are a critical component and in this capacity may incur risk to their health.

We recommend that national organizations assume responsibility for accurately tracking deaths caused by occupationally acquired infections. A nationwide tracking system will accomplish several important goals. First, it will determine the magnitude of the problem and inform future interventions. This approach has been used successfully with needlestick injuries: a problem was identified and quantified, then assessable preventive measures (e.g., the safer needle system) were put into place. This system could also lead to appropriate financial compensation. In 1976, the Public Safety Officers' Benefits Program was initiated to provide a 1-time financial benefit to survivors of police officers, firefighters, and emergency workers killed in the line of duty. The benefit also extends to those who become permanently and totally disabled as the result of trauma sustained in the line of duty.

This approach was invoked recently as the national smallpox vaccination plan was initiated. Because of healthcare worker reluctance to accept a vaccine known to cause fatal reactions, if only rarely, the government opted to extend this program to cover healthcare workers for this complication. This decision was a good start in acknowledging the unique occupational hazards of healthcare but should not remain an isolated decision made at a politically charged moment.

Most of all, a national registry would provide an ongoing reminder of the risk of caring for others, by raising awareness among laypersons and professionals alike. The 9 million persons employed in the healthcare industry and their families merit better protection for their health and greater recognition for their contributions.

## References

[1] Zuger A, Miles SH. Physicians, AIDS, and occupational risk. Historic traditions and ethical obligations. JAMA. 1987;258:1924–8. 10.1001/jama.1987.03400140086030 3309386

[2] World Health Organization. Summary of probable SARS cases with onset of illness from 1 November 2002 to 31 July 2003. 2003 Sep 26 [cited 2005 Apr 28]. Available from http://www.who.int/csr/sars/country/table2003_09_23/en/

[3] Lee N, Hui D, Wu A, Chan P, Cameron P, Joynt GM, A major outbreak of severe acute respiratory syndrome in Hong Kong. N Engl J Med. 2003;348:1986–94. 10.1056/NEJMoa030685 12682352

[4] Lew TW, Kwek TK, Tai D, Earnest A, Loo S, Singh K, Acute respiratory distress syndrome in critically ill patients with severe acute respiratory syndrome. JAMA. 2003;290:374–80. 10.1001/jama.290.3.374 12865379

[5] Zhao Z, Zhang F, Xu M, Huang K, Zhong W, Cai W, Description and clinical treatment of an early outbreak of severe acute respiratory syndrome (SARS) in Guangzhou, PR China. J Med Microbiol. 2003;52:715–20. 10.1099/jmm.0.05320-0 12867568

[6] Booth CM, Matukas LM, Tomlinson GA, Rachlis AR, Rose DB, Dwosh HA, Clinical features and short-term outcomes of 144 patients with SARS in the greater Toronto area. JAMA. 2003;289:2801–9. 10.1001/jama.289.21.JOC30885 12734147

[7] Fowler RA, Lapinsky SE, Hallett D, Detsky AS, Sibbald WJ, Slutsky AS, Critically ill patients with severe acute respiratory syndrome. JAMA. 2003;290:367–73. 10.1001/jama.290.3.367 12865378

[8] Sepkowitz KA. Occupationally acquired infections in health care workers. Part II. Ann Intern Med. 1996;125:917–28. 8967673 10.7326/0003-4819-125-11-199612010-00008

[9] Sepkowitz KA. Occupationally acquired infections in health care workers. Part I. Ann Intern Med. 1996;125:826–34. 8928990 10.7326/0003-4819-125-10-199611150-00007

[10] Bolyard EA, Tablan OC, Williams WW, Pearson ML, Shapiro CN, Deitchmann SD. Guideline for infection control in healthcare personnel, 1998. Hospital Infection Control Practices Advisory Committee. Infect Control Hosp Epidemiol. 1998;19:407–63. 10.1086/647840 9669622

[11] Mahoney FJ, Stewart K, Hu H, Coleman P, Alter MJ. Progress toward the elimination of hepatitis B virus transmission among health care workers in the United States. Arch Intern Med. 1997;157:2601–5. 10.1001/archinte.1997.00440430083010 9531229

[12] US Department of Labor, Bureau of Labor Statistics. Injuries, illnesses, and fatalities. 2002 [cited 2005 Apr 18]. Available from http://www.bls.gov/iif/home.htm

[13] US Department of Labor. Occupational Safety & Health Administration. Reporting fatalities and multiple hospitalization incidents to OSHA—1904.39. 2001 Jan 19 [cited 2005 Apr 18]. Available from http://www.osha.gov/pls/oshaweb/owadisp.show_document?p_table=STANDARDS&p_id=12783

[14] Worker Health Chartbook. 2000. 2002 May [cited 2005 Apr 18]. Available from http://www.cdc.gov/niosh/00-127pd.html

[15] Alvarado FPA, Cardo D. NaSH Surveillance Group. Percutaneous injury reporting in US hospitals, 1998 [abstract P-S2-38]. In: 4th Decennial International Conference on Nosocomial and Healthcare-associated Infections. 2000 Mar 5–9; Atlanta, Georgia.

[16] US Department of Labor. National employment and wage data from the Occupational Employment Statistics survey by occupation, November 2003. 2004 Nov 12 [cited 2005 Apr 18]. Available from http://www.bls.gov/news.release/ocwage.t01.htm

[17] Department of Labor and Bureau of Labor Statistics: Current Population Survey. 2002.

[18] National EMS Memorial Service. Notices of line of duty deaths, 2002. [cited 2005 Apr 27]. Available from http://nemsms.org/notices02.htm

[19] Centers for Disease Control and Prevention. Prevention and control of infections with hepatitis viruses in correctional settings. MMWR Recomm Rep. 2003;52(RR-1):1–36. 12562146

[20] Centers for Disease Control and Prevention. Recommendations for prevention and control of hepatitis C virus (HCV) infection and HCV-related chronic disease. MMWR Recomm Rep. 1998;47(RR-19):1–39. 9790221

[21] Alter MJ, Kruszon-Moran D, Nainan OV, McQuillan GM, Gao F, Moyer LA, The prevalence of hepatitis C virus infection in the United States, 1988 through 1994. N Engl J Med. 1999;341:556–62. 10.1056/NEJM199908193410802 10451460

[22] Henderson DK. Managing occupational risks for hepatitis C transmission in the health care setting. Clin Microbiol Rev. 2003;16:546–68. 10.1128/CMR.16.3.546-568.2003 12857782 PMC164218

[23] Gerberding JL. Clinical practice. Occupational exposure to HIV in health care settings. N Engl J Med. 2003;348:826–33. 10.1056/NEJMcp020892 12606738

[24] Rosenberg SD, Goodman LA, Osher FC, Swartz MS, Essock SM, Butterfield MI, Prevalence of HIV, hepatitis B, and hepatitis C in people with severe mental illness. Am J Public Health. 2001;91:31–7. 10.2105/AJPH.91.1.31 11189820 PMC1446494

[25] Do AN, Ciesielski CA, Metler RP, Hammett TA, Li J, Fleming PL. Occupationally acquired human immunodeficiency virus (HIV) infection: national case surveillance data during 20 years of the HIV epidemic in the United States. Infect Control Hosp Epidemiol. 2003;24:86–96. 10.1086/502178 12602690

[26] Serious adverse events attributed to nevirapine regimens for post-exposure prophylaxis after HIV exposures—worldwide, '97–'00. HIV Clin. 2001;13:11. 11810822

[27] Aoun H. When a house officer gets AIDS. N Engl J Med. 1989;321:693–6. 10.1056/NEJM198909073211020 2770800

[28] Sepkowitz KA. Tuberculosis and the health care worker: a historical perspective. Ann Intern Med. 1994;120:71–9. 8250459 10.7326/0003-4819-120-1-199401010-00012

[29] Telzak EE, Sepkowitz K, Alpert P, Mannheimer S, Medard F, el-Sadr W, Multidrug-resistant tuberculosis in patients without HIV infection. N Engl J Med. 1995;333:907–11. 10.1056/NEJM199510053331404 7666876

[30] Sepkowitz KA. AIDS, tuberculosis, and the health care worker. Clin Infect Dis. 1995;20:232–42. 10.1093/clinids/20.2.232 7742421

[31] Weltman AC, DiFerdinando GT Jr, Washko R, Lipsky WM. A death associated with therapy for nosocomially acquired multidrug-resistant tuberculosis. Chest. 1996;110:279–81. 10.1378/chest.110.1.279 8681641

[32] Munsiff SS, Nivin B, Sacajiu G, Mathema B, Bifani P, Kreiswirth BN. Persistence of a highly resistant strain of tuberculosis in New York City during 1990–1999. J Infect Dis. 2003;188:356–63. 10.1086/376837 12870116

[33] Centers for Disease Control and Prevention update: adverse event data and revised American Thoracic Society/CDC recommendations against the use of rifampin and pyrazinamide for treatment of latent tuberculosis infection—United States, 2003. MMWR Morb Mortal Wkly Rep. 2003;52:735–9. 12904741

[34] Sagoe-Moses C, Pearson RD, Perry J, Jagger J. Risks to health care workers in developing countries. N Engl J Med. 2001;345:538–41. 10.1056/NEJM200108163450711 11519511

[35] Harries AD, Nyirenda TE, Banerjee A, Boeree MJ, Salaniponi FM. Tuberculosis in health care workers in Malawi. Trans R Soc Trop Med Hyg. 1999;93:32–5. 10.1016/S0035-9203(99)90170-0 10492785

[36] Eyob G, Gebeyhu M, Goshu S, Girma M, Lemma E, Fontanet A. Increase in tuberculosis incidence among the staff working at the Tuberculosis Demonstration and Training Centre in Addis Ababa, Ethiopia: a retrospective cohort study (1989–1998). Int J Tuberc Lung Dis. 2002;6:85–8. 11931406

[37] Wilkinson D, Gilks CF. Increasing frequency of tuberculosis among staff in a South African district hospital: impact of the HIV epidemic on the supply side of health care. Trans R Soc Trop Med Hyg. 1998;92:500–2. 10.1016/S0035-9203(98)90889-6 9861361

[38] Gumodoka B, Favot I, Berege ZA, Dolmans WM. Occupational exposure to the risk of HIV infection among health care workers in Mwanza Region, United Republic of Tanzania. Bull World Health Organ. 1997;75:133–40. 9185365 PMC2486935

[39] The Officer Down Memorial Page, Inc. Honoring officers killed in the year 2002. [cited 2005 May 2]. Available from http://www.odmp.org/year.php?year=2002&Submit=Go

[40] United States Fire Administration. USFA firefighter fatalities. 2001 [cited 2005 Apr 18]. Available from http://www.usfa.fema.gov/inside-usfa/nfdc/pubs/ff_fat.shtm

